# Uncovering critical properties of the human respiratory syncytial virus by combining *in vitro* assays and *in silico* analyses

**DOI:** 10.1371/journal.pone.0214708

**Published:** 2019-04-15

**Authors:** Catherine A. A. Beauchemin, Young-In Kim, Qin Yu, Giuseppe Ciaramella, John P. DeVincenzo

**Affiliations:** 1 Department of Physics, Ryerson University, Toronto, Ontario, Canada; 2 Interdisciplinary Theoretical and Mathematical Sciences (iTHEMS) Research Program at RIKEN, Wako, Saitama, Japan; 3 Department of Pediatrics, University of Tennessee Health Science Center, Memphis, Tennessee, United States of America; 4 Children’s Foundation Research Institute at Le Bonheur Children’s Hospital, Memphis, Tennessee, United States of America; 5 AstraZeneca Pharmaceuticals, Waltham, Massachusetts, United States of America; 6 Department of Microbiology, Immunology and Biochemistry, University of Tennessee Health Science Center, Memphis, Tennessee, United States of America; University of Iowa, UNITED STATES

## Abstract

Many aspects of the respiratory syncytial virus (RSV) are still poorly understood. Yet these knowledge gaps have had and could continue to have adverse, unintended consequences for the efficacy and safety of antivirals and vaccines developed against RSV. Mathematical modelling was used to test and evaluate hypotheses about the rate of loss of RSV infectivity and the mechanisms and kinetics of RSV infection spread in SIAT cells *in vitro*. While the rate of loss of RSV integrity, as measured via qRT-PCR, is well-described by an exponential decay, the latter mechanism failed to describe the rate at which RSV A Long loses infectivity over time in vitro based on the data presented herein. This is unusual given that other viruses (HIV, HCV, influenza) have been shown to lose their infectivity exponentially *in vitro*, and indeed an exponential rate of loss of infectivity is always assumed in mathematical modelling and experimental analyses. The infectivity profile of RSV in HEp-2 and SIAT cells remained consistent over the course of an RSV infection, over time and a large range of infectivity. However, SIAT cells were found to be ∼ 100× less sensitive to RSV infection than HEp-2 cells. In particular, we found that RSV spreads inefficiently in SIAT cells, in a manner we show is consistent with the establishment of infection resistance in uninfected cells. SIAT cells are a good *in vitro* model in which to study RSV *in vivo* dissemination, yielding similar infection timescales. However, the higher sensitivity of HEp-2 cells to RSV together with its RSV infectivity profile being similar to that of SIAT cells, makes HEp-2 cells more suitable for quantifying RSV infectivity over the course of *in vitro* RSV infections in SIAT cells. Our findings highlight the importance and urgency of resolving the mechanisms at play in the dissemination of RSV infections *in vitro*, and the processes by which this infectivity is lost.

## Introduction

Careful mathematical analysis of viral infection kinetics can provide valuable insight into important features or particularities of a virus which might not be identifiable from the observation of experimental data alone. In some cases, inferences based on experimental observations alone can be misleading. For example, the observed slow progression from HIV infection to AIDS suggests slow cell and virus turnover, yet a mathematical analysis of the plasma viral load decay under antiviral therapy revealed the very rapid kinetics of production matched by an equally rapid suppression by host immunity [1–6]. This finding meant that a very different strategy was required for optimal antiviral therapy, one that takes into consideration the higher likelihood of antiviral resistance emergence brought about by the higher virus production rates than initially believed.

The human respiratory syncytial virus (RSV) is an important respiratory pathogen and like many viruses, its control has posed important challenges. To date, no vaccine or antiviral therapy is approved to prevent or control RSV infection. The development of safe, reliable and effective anti-RSV vaccine and antiviral therapy would benefit tremendously from a more in-depth understanding of the particularities of RSV in terms of what characterizes its infectivity and cell-to-cell dissemination.

It has long been known that RSV is an unstable virus which undergoes rapid inactivation, quickly losing infectivity [7–12]. Infectivity is, at least in part, affected by the conformation of the RSV fusion (F) surface protein which can deform, non-reversibly, from a pre- to a post-trigger conformation. It has recently been shown that a decrease in the number of virion-associated, F proteins in the pre-trigger conformation correlates highly with a decrease in the virions’ ability to cause infection [13]. Additionally, it is known that low molarity (low ionic/salt concentration) triggers a change in the conformation of RSV’s F proteins from the pre- to the post-trigger state [14–16], and salt is known to help stabilize RSV and slow down the rate of infectivity loss [11]. Without knowing which process drives the loss of RSV infectivity, it is difficult to decide how best to capture the process mathematically.

Herein, we experimentally investigate a number of key aspects of RSV: its distinctive rate of loss of infectivity over time, the change in its infectivity profile in different cell types, and the peculiar kinetics of its dissemination from cell-to-cell. We make use of various mathematical models (MMs) to uncover the mechanisms behind the trends observed in the experimental data. In doing so, we identify a number of important gaps in understanding the processes involved in RSV infection and their possible implications for the development and optimization of preventive and therapeutic RSV interventions.

## Results

### Understanding how respiratory syncytial virions lose infectivity

**Fig 1:**
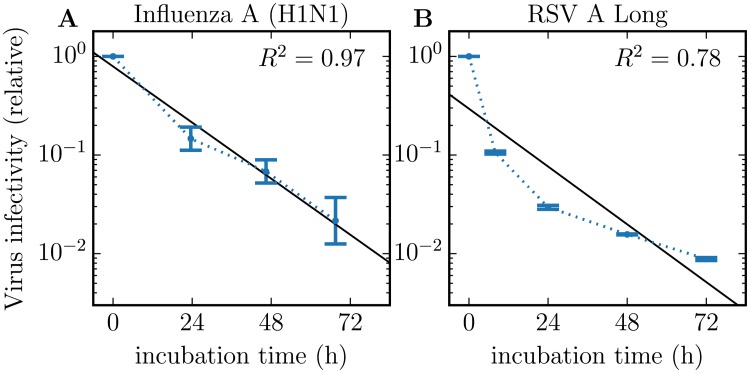
Rate of loss of virion infectivity. Comparison of the rate of loss of virus infectivity at 37°C for (A) influenza A (H1N1) virus (A/New Caledonia/20/1999-like clinical isolate) from [17] against that for (B) the respiratory syncytial virus (RSV A Long) from the present work (blue dots joined by dotted blue line). While the loss of infectivity by influenza A virions follows an exponential (straight black line), that of RSV does not appear to, yielding a lower *R*^2^ value. The dots and error bars represent the geometric means and standard deviations of 3 biological replicates (influenza) or 2 measurement duplicates from a single biological experiment (RSV).

The loss of infectivity of different viruses (influenza, HIV, hepatitis C) *in vitro* has been shown to follow an exponential decay [17–19](HCV-JFH1, unpublished data, Dr. Koichi Watashi, National Institute of Infectious Diseases, Tokyo, Japan). This implies that individual virions have an equal probability of losing infectivity every second, irrespective of how much time has elapsed since they were produced and released by an infected cell. Fig 1 compares the rate of loss of infectivity of RSV against that of an influenza A virus strain *in vitro*. While the rate of infectivity loss for the influenza A virus follows an exponential decay, that of RSV seemingly does not. Instead, the rate of loss of RSV infectivity appears slows down over time.

To explore the rate of infectivity loss of RSV, we conducted a mock-yield (MY) assay in which RSV virions were incubated in medium in the absence of cells, and the concentration of total and infectious RSV remaining as a function of incubation time was quantified. Here, the total virus concentration refers to the concentration of RSV virions which have retained sufficient genetic integrity to be amplified and thus quantified via quantitative, real-time, reverse-transcription, polymerase chain reaction (qRT-PCR, see Methods), providing a measure that is proportional to the concentration of RSV viral RNA (viral RNA equivalent or vRNAe hereafter). As for the infectious virus concentration, it refers to the concentration of RSV virions which have retained their ability to cause infection, which is quantified herein using both units of PFU in HEp-2 cells and TCID_69_ in SIAT cells (further explanation of TCID_69_ is provided in Methods). Biologically, virions first lose infectivity and eventually will lose vRNA integrity such that the rate of loss of infectious virus is invariably more rapid than the rate of loss of total virus.

**Fig 2:**
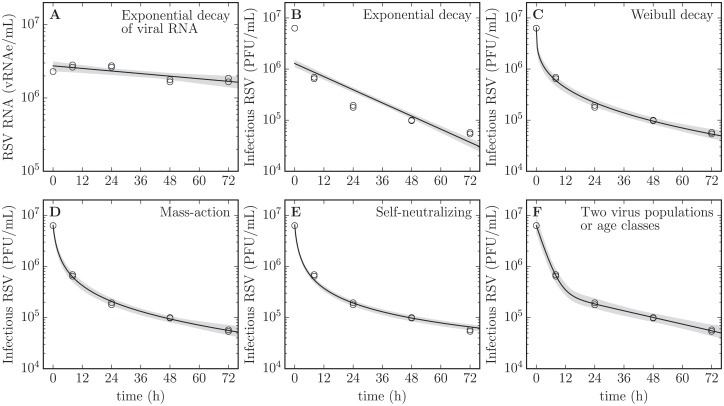
Rate of loss of RSV integrity and infectivity in a mock-yield assay. Various MM expressions, each implying different mechanisms, are considered to try and determine which best reproduces the observed kinetics of RSV integrity (A) and infectivity (B–F) loss over time at 37°C, quantified in duplicate. The grey band represents the variation in the predicted decay shape based on the estimated 95% credible region for the MM parameters. Refer to the text for the biological meaning and implications of each MM. Duplicate measures (two symbols) rather than error bars are plotted at each sampling time points, though in some cases only one point is visible.

In Fig 2, the experimentally measured, remaining concentration of total and infectious RSV over time is presented against various MMs meant to capture their decay rates. The performance of these different MMs in the context of our RSV MY data is presented quantitatively in Table 1. For each MM, whose biological interpretation is explained below, a Markov chain Monte Carlo (MCMC) process was used to parametrize the MMs to our MY data, and estimate the posterior probability likelihood distributions (PostPLDs) of the parameters characterizing each MM. In Table 1, the sum-of-squared residuals (SSR) provides a measure of the goodness-of-fit of each MM, with smaller SSRs suggesting better fits (smaller residuals). Since the different MMs considered contained differing number of parameters; the SSR must be corrected to account for the additional degrees of freedom of MMs with more parameters. Akaike’s Information Criterion corrected for small sample sizes (AIC_C_) accounts for this and provides a measure of the goodness of fit that can be compared across the different MMs, with a lower AIC_C_ (e.g., -25 better than -10) identifying MMs that best balance goodness of fit and number of parameters. These different MMs each represent different biological assumptions about the manner or process by which RSV virions lose integrity (total virions, vRNAe) or infectivity (infectious virions, PFU or TCID_69_), and we will now explain each in turn.

**Table 1:** Performance of various MMs for RSV loss of infectivity*.

Model (Eq#), *V*(*t*) = …	SSR [95%CR]	AIC_C_ [95%CR]	Parameter values [95%CR]
Exponential (2), *V*_0_ exp [−*ct*] [viral RNA]	0.204 [0.19, 0.24]	-23.3 [-24, -22]	*c* = 0.0068 [0.0042, 0.01]
Exponential (2), *V*_0_ exp [−*ct*]	0.894 [0.89, 0.90]	-9.98 [-10, -9.9]	*c* = 0.049 [0.046, 0.053]
Weibull (3), *V*_0_ exp [−(*ct*)^*k*^]	0.166 [0.14, 0.22]	-17.9 [-20, -15]	*c* = 2.3 [1, 5],
			*k* = 0.3 [0.27, 0.36]
Mass-action (4), $\frac{\left(1-f\right)V_{0}}{1-f\text{exp}[-(1-f)ct]}$	0.132 [0.094, 0.19]	-20 [-23, -17]	*c* = 1.1 [0.78, 1.6],
			*f* = 1 [1, 1]
Self-neutralizing (5), $\frac{V_{0}}{1+ct}$	0.183 [0.17, 0.22]	**-24.3 [-25, -22]**	*c* = 1.4 [1.1, 1.9]
Two populations (6), $V_{0}\left[f_{\text{low}}{\text{e}}^{-c_{\text{low}}t}+(1-f_{\text{low}}){\text{e}}^{-c_{\text{high}}t}\right]$	0.120 [0.044, 0.19]	-8.87 [-18, -4.6]	*c*_low_ = 0.025 [0.02, 0.032],
			*c*_high_ = 0.34 [0.29, 0.41],
			*f*_low_ = 0.055 [0.036, 0.082]
Two age classes (7), $V_{0}e^{-r_{\text{pre}}t}[1-\frac{r_{\text{post}}}{r_{\text{pre}}-c_{\text{low}}}(1-e^{(r_{\text{pre}}-c_{\text{low}})t})]$	0.120 [0.044, 0.19]	-8.87 [-18, -4.6]	*c*_low_ = 0.025 [0.02, 0.032],
			*r*_pre_ = 0.34 [0.29, 0.41],
			*r*_post_ = 0.017 [0.011, 0.027]

* The sum of squared residuals (SSR) provides a quantitative measure of the best fit to the data irrespective of the number of parameters in the MM. Akaike’s “An Information Criterion” corrected for small samples (AIC_C_) provides a measure of the best MM by penalizing MMs with additional parameters. The smaller these numbers, the more suitable the MM given the data. The median SSR, AIC_C_ and parameter values are provided along with the 95% credible region (CR) for the latter.

Rates of loss of total and infectious virus are typically faithfully captured by the exponential decay MM (Fig 2A and 2B). The exponential decay MM assumes each virion has an equal probability of losing integrity (vRNAe) or infectivity (PFU or TCID_69_) every second, irrespective of how much time has elapsed since its production and release from infected cells or since the start of the incubation period. An exponential rate of decay appears as a straight line (linear relationship) on a logarithmic-vs-linear plot, like that used in Figs 1 and 2. The rate of loss of total RSV, i.e. degradation of vRNA integrity, is well-described by an exponential decay, exhibiting the clear linear trend expected for such a decay. The rate of loss of infectious RSV, however, does not appear consistent with an exponential decay, providing the worst agreement (highest SSR and AIC_C_) out of all other MMs considered below. This is because the 10-fold loss of infectivity observed over the first 8 h of incubation is the same as that observed between 8 h and 48 h of incubation, suggesting a significant slow-down in the rate of loss of infectivity over time. This is inconsistent with an exponential rate of decay of RSV infectivity.

This apparent deviation from the exponential was further investigated using a Weibull survival function (Fig 2C). The Weibull survival function, which is the complementary cumulative distribution function of the Weibull distribution, has a shape parameter (*k*) which is such that if *k* = 1, the rate of loss is constant over time, resulting in exponential decay. A *k* > 1 corresponds to a rate of loss which increases over time, such as would result from accumulated wear-and-tear or aging, leading to eventual failure. A *k* < 1 corresponds to a decreasing rate of loss over time. Via our MCMC Bayesian approach, we determined that the Weibull shape parameter for our RSV MY data is *k* = 0.3 (95% credible region or 95%CR [0.27, 0.36]), statistically significantly less than one and therefore clearly not exponential. In the manufacturing sector, wherefrom the Weibull function originates, this type of failure is typically indicative of a low-quality production process with many early failures of defective products shortly after production, and a decreasing failure rate over time as the defective elements eliminate themselves from the population; an interesting analogy we shall return to in the Discussion.

There exist several other MMs to describe the loss of viral infectivity, each pointing to different mechanisms by which infectivity is lost. Many such processes for various viruses and MMs were discussed in an excellent paper by Hiatt in 1964 [20], and some of them are explored in Fig 2 and Table 1. The mass-action MM (Fig 2D) implies that virions’ infectivity is neutralized by some agent, present in finite quantity in the medium, which gets consumed as more virions are neutralized. The self-neutralizing MM (Fig 2E) implies that virions are neutralizing themselves such that as more virions are neutralized, there are fewer virions available to neutralize those remaining. This MM could therefore represent the infectivity neutralization that results from the aggregation of virus in the RSV stock. The two virus population MM (Fig 2F) assumes that virions come in two types, each with its exponential rate of infectivity loss. The two age class MM (mathematically equivalent to Fig 2F) suggests that virions are initially in a highly infectious conformation, decay exponentially into a less infectious conformation, from which they ultimately decay as they lose all infectivity.

Of all the MMs considered here to describe the RSV rate of infectivity loss, the exponential MM provides the worst agreement (highest SSR) compared to all other MMs (all *p*-values < 10^−6^, with all *p*-values reported in this work computed as described in Methods), but all other MMs perform equivalently well in that regard (all *p*-values ≥ 0.06). Since the different MMs range from having 2–4 free parameters, the balance of goodness-of-fit to parsimony, measured by the AIC_C_, varies significantly between MMs: the 1-parameter self-neutralization MM performed best (lowest AIC_C_, all *p*-values < 0.009), followed by the 2-parameter mass-action and Weibull MMs, with the two population and two age class 4-parameter MMs doing worst. The two populations and two age class MMs are mathematically equivalent which is why they yield essentially the same parameters in Table 1. Whereas the two population MM assumes two distinct populations of virus with different infectivity profiles, the MM with two age classes assumes that the two populations correspond to two different stages traversed by RSV after their production, with a first highly infectious state followed by one of lesser infectivity, and finally a complete loss of infectivity.

In our analysis of RSV infection kinetics below, we describe RSV loss of infectivity using the two age class MM. It was chosen because, while it did not perform as well AIC_C_-wise as other alternative MMs evaluated, it is most easily implemented mathematically in an infection context. For example, the Weibull MM requires keeping track of the age of individual virions, which is difficult to implement in our existing infection MM. The mass-action and self-neutralization MMs requires an understanding of how virus concentration (or concentration of the unknown neutralizing agent) modulates the infectivity decay rate. While the analysis in Table 1 identifies this rate for the specific virus concentration in the infectivity assay, the assay and analysis do not provide sufficient information to forecast how that rate would change for higher or lower virus concentrations, say over the course of an infection, or as media is replenished which could replenish the unidentified neutralizing agent.

### Measures of virus infectivity in SIAT versus HEp-2 cells

In vitro study of RSV infections have been conducted on HEp-2, A549, Vero, MDCK, primary paediatric bronchial epithelial cells (PBECs), human airway epithelium (HAE) [21–27]. While various cell cultures have their respective merit; the aim is to use a cell culture which most closely recapitulates the mechanisms of spread, infectivity profile, and kinetics of an in vivo RSV infection. This impetus, however, must be weighted against the need for an infection system which yields robust, reproducible results; something difficult to achieve in primary cell lines. We elected to conduct our RSV in vitro infection experiment on SIAT cells. SIAT cells are modified Madin Darby canine kidney (MDCK) cells which have been transfected to express greater quantities of *α*-2, 6 sialic acid receptors on their surface. SIAT cells are commonly used in studies of influenza A virus infections in vitro, and while they have not previously been used to study RSV, MDCK cells have been [21, 25, 27]. For RSV, heparan sulfate proteoglycans (HSPGs), rather than sialic acid receptors used by influenza A virus, are believed to play a key role in cell attachment *in vitro* [28].

In addition to the cell-free, mock-yield (MY) assay discussed in the previous section, we conducted three separate RSV *in vitro* infection experiments in SIAT cells, each initiated with different multiplicities of infection (MOI) or dilutions of the RSV inoculum, infecting a small fraction of cells to observe the kinetics of multiple rounds of RSV replication through the SIAT cell culture. We will return to the analysis of these infections in the next section.

First, to specifically investigate the infectivity profile of RSV, we quantified the concentration of infectious extracellular RSV in the supernatant sampled frequently over the course of the infection of SIAT cells. Each supernatant sample was quantified both via PFU on SIAT cells and via TCID_69_ in HEp-2 cells. Quantification of infectious virus in SIAT cells was done to quantify on the same cell type as the infection itself (which was only ever conducted on SIAT cells). Additional quantification on HEp-2 cells was done to ensure that any low titers, which could not be quantified on the less sensitive SIAT cells, could still be reliably quantified on HEp-2. If SIAT are simply less sensitive to infection than HEp-2, the RSV infectivity ratio of PFU on SIAT to TCID_69_ on HEp-2 should be consistent; a consistent ratio of 1:100 would indicate SIAT are 100× less sensitive to RSV infection than HEp-2. In contrast, finding that this ratio is not consistent over time or between experiments (i.e. statistically significantly different beyond inter-experimental variability) would indicate a difference in what characteristics make RSV infectious in these 2 cell lines. For example, if both spherical and filamentous RSV morphologies were equally infectious on HEp-2 but only the former was infectious on SIAT, changes in the ratios of these two morphologies over time or between experiments would yield inconsistent infectivity ratios which would be detectable if the effect is statistically significant.

**Fig 3:**
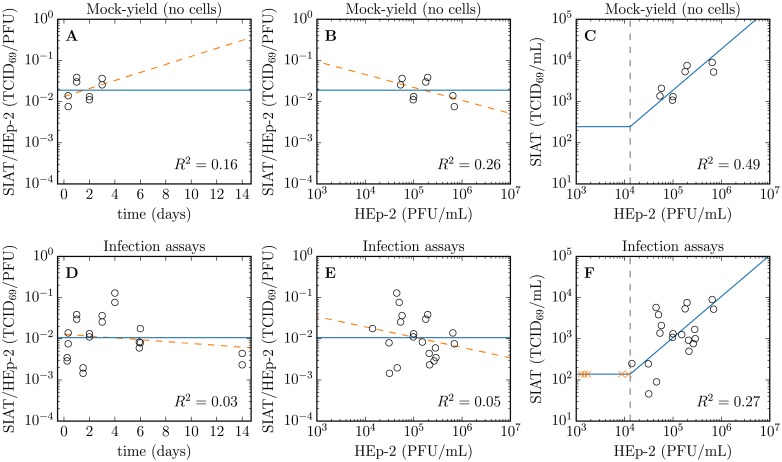
Measures of RSV infectivity on SIAT and HEp-2 cells. Comparison of the infectivity of individual RSV samples as measured on SIAT cells in TCID_69_/mL compared to that measured on HEp-2 cells in PFU/mL for each sample. The SIAT/HEp-2 infectivity ratio is shown (A) as a function of incubation time in the mock-yield (no cells) assay; or (D) as a function of infection time in the infection assays; or (B, E) as a function of the sample’s infectivity when quantified on HEp-2 cells. The solid horizontal line in (A, B, D, E) corresponds to the geometic (logarithmic) mean of the infectivity ratio (SIAT/HEp-2), and the comparative dashed line corresponds to a log-lin (A, D) or log-log (B, E) linear regression with associated *R*^2^ value provided. (C, E) RSV sample infectivity quantified on SIAT cells is shown as a function of that on HEp-2 cells in the mock-yield (C) and infection (F) assays. The solid diagonal line indicates the expected relationship between SIAT and HEp-2 and points should fall along this line. The ‘×’ denote samples with a measurable infectivity on HEp-2 cells (their *x*-value) that was undetectable on SIAT cells. These points were placed at what appears to be the limit of infectivity detection in SIAT cells, which corresponds to an infectivity of 1.3 × 10^4^ PFU/mL in HEp-2 cells, indicated by a vertical dashed line. The data shown here are the same as those shown again as a function of time in Figs 2 and 4.

Fig 3 compares quantification of RSV infectivity on SIAT via TCID_69_ with that on HEp-2 via PFU. Fig 3(A) and 3(D) show that the ratio of SIAT/HEp-2 infectivity (TCID_69_/PFU) appears to be time-independent, i.e. does not appear to vary in a specific direction over time systematically. This suggests that both SIAT and HEp-2 are likely susceptible to the same aspects of whatever makes RSV infectious. In particular, this appears to hold true as the infectivity profile of the virus population shifts over time as their infectivity decreases. In other words, the rate of loss of RSV infectivity appears to be similar in either cell types. Fig 3(B) and 3(E) explores whether the ratio of SIAT/HEp-2 infectivity depends on infectivity itself rather than on time, i.e. whether a population of highly infectious RSV or relatively uninfectious RSV would exhibit the same relative infectivity profile (infectivity ratio) in the two cell types. Again, the SIAT/HEp-2 infectivity ratio does not appear to vary systematically in a particular direction as a function of infectivity itself. Looking more closely at samples which had a quantifiable infectivity in HEp-2 but not in SIAT cells, Fig 3(C) and 3(F) suggests that samples with an infectivity of ∼10^4^ PFU/mL or less in HEp-2 were not quantifiable in SIAT cells. In contrast with the MY experiment (Fig 3A–3C) which observes virus infectivity over time in the absence of cells, RSV kinetics in infection experiments (Fig 3E and 3F) does appear to result in more varying SIAT/HEp-2 infectivity ratios, likely owing to the larger variability inherent in infection experiments.

### Characterizing *in vitro* RSV infection kinetics using *in silico* models

**Fig 4:**
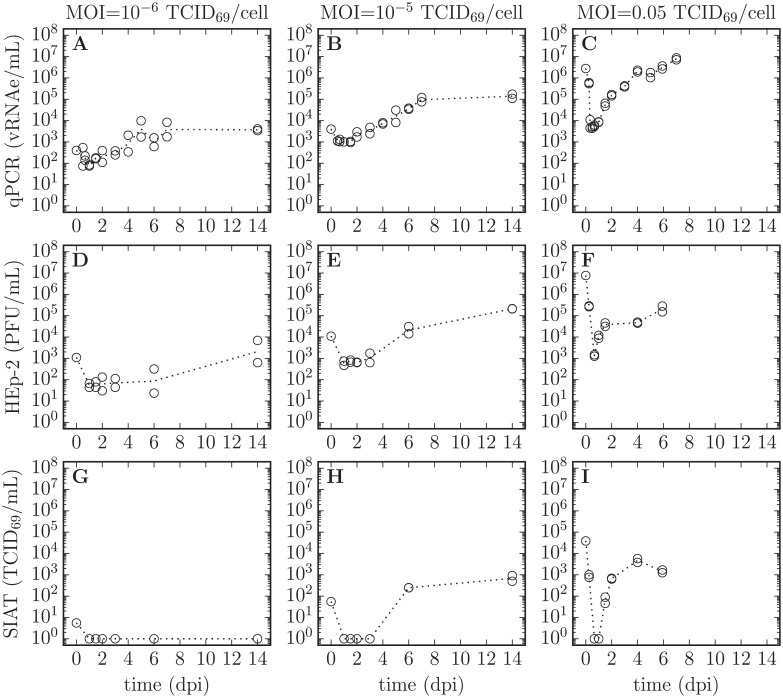
Time course of *in vitro* RSV infection in SIAT cells. SIAT cells were infected in duplicate with 3 different dilutions (MOIs) of an RSV A Long inoculum. Over the course of the *in vitro* RSV infections, the supernatant was sampled multiple times to establish RSV infection kinetics. The total RSV concentration was measured via qRT-PCR (vRNAe/mL, A, B, C), whereas infectious RSV concentration was quantified both on HEp-2 cells (PFU/mL, D, E, F) and on SIAT cells (TCID_69_/mL, G, H, I). Duplicate measures (two symbols) rather than error bars are plotted at each sampling time point, though in some cases only one point is visible. A dashed line joins the geometric mean of the measures at each sampling time point.

The infection experiments were conducted in SIAT cells using 3 different RSV infection inocula, one of high concentration, and two of low concentration. At each sampling time, the supernatant in two duplicate wells was harvested, and the concentration of total and infectious RSV in each of the two supernatant samples was determined. No sampling well was re-used; each supernatant sample was harvested from a distinct well and the well was then discarded. Cell-associated or intracellular virus was not sampled. Total RSV was quantified via quantitative, real-time, reverse transcriptase PCR (hereafter qRT-PCR, in vRNAe/mL) and infectious RSV was quantified on HEp-2 cells (in PFU/mL) and SIAT cells (in TCID_69_/mL), as described in Methods. The data for these RSV infection experiments are presented in Fig 4.

**Fig 5:**
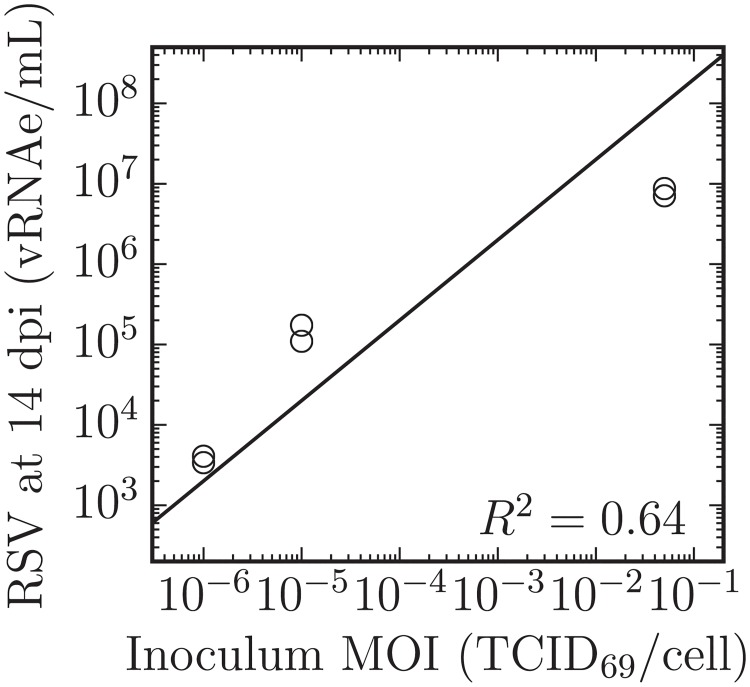
Total RSV production is proportional to the inoculum MOI. The concentration of total RSV in the supernatant quantified via qRT-PCR (vRNAe/mL) at 14 dpi for infections performed in duplicate is shown as a function of the MOI of the inoculum deposited onto the SIAT cells (TCID_69_/cell). The relation shown (solid line) corresponds to log_10_(*y*) = log_10_(*x*) + *b* where *b* = 9.3 ± 0.8 and *R*^2^ is as shown. The total number of RSV progeny produced over the course of the infections is approximately proportional to inoculating MOI. Duplicate measures (two symbols) rather than error bars are plotted at each sampling time point, through in some cases only one point is visible.

There are a number of interesting features visible in the RSV infection data presented in Fig 4. The total RSV concentration (infectious + non-infectious virions) as quantified via qRT-PCR appears to reach a plateau, remaining seemingly unchanged between the samples harvested at 7 dpi and 14 dpi. Interestingly, this plateau is approximately proportional to the input inoculum, as shown in Fig 5. Assuming an RSV-infected cell will always produce a certain number of virions, the total number of virions produced (the height of this plateau) should be proportional to the number of cells that became infected over the course of the infection. This would mean that only the RSV inoculum caused infection and the RSV progeny produced *de novo* during the infection process resulted in a negligible number of infection events.

Unlike the total RSV concentration, the infectious RSV concentration (TCID_69_/mL) appears to continue to increase, rather than plateau, between the samples harvested at 6 dpi and 14 dpi. For example, for the infection initiated with a MOI of 10^-5^ TCID_69_/cell, RSV titer rises from (246, 249) TCID_69_/mL at 6 dpi to (497, 919) TCID_69_/mL at 14 dpi. An increase in infectious RSV titer would contradictorily suggest that the RSV progeny produced *de novo* during the infection process can cause productive infections in SIAT cells beyond those caused by the inoculum.

Taken together, these observations suggest that either RSV has a low but non-negligible infectivity activity in SIAT cells, or that the infectious virus produced by the RSV-infected SIAT cells is non-infectious within the wells in which the infection is proceeding, but is infectious when placed in fresh, uninfected SIAT cells. This could be explained, for example, by the activation of an antiviral state in uninfected cells which would arise shortly after the first cycle of cells are infected by the initial inoculum. Alternatively, the cessation of infectivity in the infection assay itself but not in the plaque assay could possibly be due to the presence of defective interfering RSV that could be present in higher concentration and therefore be more potent in the infection assay than in the plaque assay. Whatever the possible cause behind the establishment of resistance to infection in the assay, it is possible to explore this additional hypothesis mathematically. In the *in silico* MM, this is done by completely blocking cell infection (setting parameter *β* = 0 in the MM, see Methods, Eq (11)) at time *τ*_*F*_ post infection, a parameter which is determined as part of fitting our MM to the data. Thus, we consider the following variations of the *in silico* MM to represent our *in vitro* RSV infections:

**Poor infection efficacy** which assumes the slow infection kinetics are the result of either poor RSV yield from SIAT cells and/or their poor sensitivity to infection by RSV; or
**Infection resistance** is activated at some time *τ*_*F*_ post infection which blocks any further infection of cells by the free virus. This process could arrest infection promptly after the first cycle of infection, and result in a total RSV concentration plateau proportional to the concentration of RSV in the initial inoculum.

**Fig 6:**
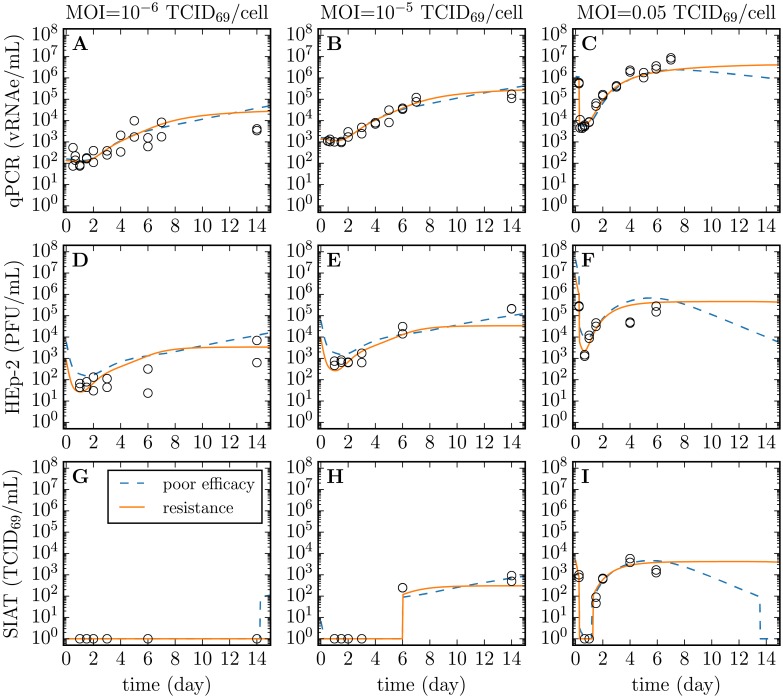
Time course of the parametrized *in silico* MM for the *in vitro* infection of SIAT cells with RSV A Long. Parameters of the *in silico* MMs (lines) Eqs (9)–(11) were estimated by simultaneously considering all data from Fig 4 (circles) for the total RSV yield quantified via qRT-PCR (A, B, C), and the RSV infectious titer quantified both on HEp-2 (D, E, F) and SIAT (G, H, I) cells, of supernatant samples collected from experimental infection of SIAT cells, performed in duplicate, at 3 different MOIs (3 different columns). The blue lines correspond to the poor infection efficacy MM, Eqs (9) and (10), while the orange line corresponds to the infection resistance MM, Eqs (9)–(11), wherein infectivity is assumed to be zero (*β* = 0) a time *τ*_*F*_ post-infection.

The mathematical details of the full *in silico* infection MMs can be found in the Methods section, Eqs (9)–(11). Within the infection MMs, the rate of loss of RSV infectivity is captured using the two age class MM, Eq (10), using the parameter values reported in Table 1. For each of the 2 hypotheses (or infection MMs) considered, namely poor infection efficacy or infection resistance, a MCMC process was used to estimate the PostPLDs of the MMs’ parameters. The mode and 95% CR of each parameter’s PostPLDs (marginalized over all other parameters) are presented in Table 2, along with the ‘best’ value, i.e. the value of the parameter within the parameter set which yielded the highest likelihood. Each parameter’s 95% CR provide a measure of the parameter’s identifiabiliy, while details regarding pair-wise parameter correlations are provided in Methods. The MM-predicted time course for the infection of SIAT cells with RSV A Long is shown in Fig 6 based on the ‘best’ parameter values, alongside the experimental *in vitro* infection data.

**Table 2:** MCMC-estimated parameter values for the infection MMs*.

Parameter (units)	Poor efficacy	Infection resistance	*p*- value
Inoculum, *V*_PCR_(0) $\left(\frac{\text{vRNA}}{\text{mL}}\right)$	10^2.2 [2.1, 2.3], (2.2)^	10^2.1 [2, 2.3], (2.1)^	0.248
Inoculum, *V*_HEp−2_(0) $\left(\frac{\text{PFU}}{\text{mL}}\right)$	10^3.8 [3.8, 3.9], (3.8)^	10^2.9 [2.6, 3.2], (2.9)^	<**0.001**
Inoculum, *V*_SIAT_(0) $\left(\frac{{\text{TCID}}_{69}}{\text{mL}}\right)$	10^−0.09 [−0.38, 0.16], (−0.17)^	10^−0.1 [−0.38, 0.17], (−0.18)^	0.490
SC rinse factor, $\frac{V_{\text{post}-\text{rinse}}}{V_{\text{pre}-\text{rinse}}}$	10^−2.3 [−2.5, −2.2], (−2.3)^	10^−2.2 [−2.4, −1.9], (−2.1)^	0.136
Prod. rate, *p*_PCR_ $\left(\frac{\text{vRNA}}{\text{mL}\cdot h}\right)$	10^4.8 [4.5, 5.6], (4.7)^	10^4.5 [4.3, 4.7], (4.5)^	**0.050**
Prod. rate, *p*_HEp−2_ $\left(\frac{\text{PFU}}{\text{mL}\cdot h}\right)$	10^5.4 [5.2, 6.2], (5.3)^	10^5.1 [4.8, 5.3], (5)^	**0.014**
Prod. rate, *p*_SIAT_ $\left(\frac{{\text{TCID}}_{69}}{\text{mL}\cdot h}\right)$	10^3.2 [3, 4], (3.1)^	10^3 [2.8, 3.2], (2.9)^	0.052
Infectiousness, *β* $\left(\frac{\text{mL}}{\text{PFU}\cdot h}\right)$	10^−7.3 [−7.4, −7.1], (−7.2)^	10^−6.4 [−6.7, −6.2], (−6.4)^	<**0.001**
Eclipse phase, *τ*_*E*_ (days)	3.7 [3.1, 5.7], (3.5)	3 [2.5, 3.5], (2.8)	**0.027**
Virus-prod. phase, *τ*_*I*_ (days)	0.63 [0.45, 4.2], (3.6)	16 [9.5, ND], (15)	<**0.001**
Antivir. timing, *τ*_*F*_ (days)	—	4.5 [3.5, 5.4], (4.5)	—
Derived (computed) parameters			
Infecting time, *t*_inf_ (h)	13 [4.9, 14], (13)	6.9 [5.4, 8.6], (7.5)	0.233
Infection rate, *R*_0_/*τ*_*I*_ (1/day)	1.1 [0.74, 7], (1.1)	3.6 [2.3, 6.1], (3.4)	0.233
Basic repro. num., *R*_0_	3.9 [3.1, 4.7], (3.9)	50 [26, ND], (50)	<**0.001**
Sum Squared Resid., SSR	240 [230, 250], (230)	180 [170, 190], (170)	<**0.001**
Akaike Inform. Crit., AIC_C_	110 [100, 110], (100)	72 [67, 80], (65)	<**0.001**

* Estimates are provided as: Mode [95% credible region] (best), where ‘best’ corresponds to the value of that parameter in the parameter set that yields the highest likelihood. A CR bound of *‘ND’* indicates the parameter is not bounded. The *‘Poor efficacy’* MM has 10 estimated parameters whereas the *‘Infection resistance’* MM has 11 (*τ*_*F*_). This was taken into account in the calculation of the AIC_*C*_. Parameters of the two age class MM for RSV infectivity loss MMs were fixed to the maximum likelihood values identified in Table 1 and thus are not included in the count of estimated parameters. The *‘Derived parameters’* were computed from the MM’s estimated parameters.

The infection resistance MM performed significantly better (*p*- value < 0.001) than the poor infection efficacy MM in terms of SSR, but also in terms of AIC_C_, i.e. even when penalized for its one additional parameter (*τ*_*F*_). In the infection resistance MM, infection progress is arrested by the unspecified resistance mechanism built into the MM whereby cells are assumed resistant to RSV infection a time *τ*_*F*_ post-infection. Fig 6 shows that this process results in, and thus can explain, the plateau in the infectious RSV titer and the slow growth in the total RSV concentration observed experimentally. In contrast, in the poor infection efficacy MM, infection progress is arrested by the death of infected cells a time *τ*_*I*_ after they begin producing and releasing RSV, and this results in a peak followed by a decay in the total and infectious RSV concentrations.

The two different explanations offered by these MMs for the observed infection kinetics result in some differences in parameter estimates. In the poor infection efficacy MM, this inefficacy is captured via an 18 h longer delay or eclipse phase (*τ*_*E*_) prior to the start of virus production by newly infected cells, followed by a much shorter period of virus production (*τ*_*I*_) by these cells. Over its short 15 h period of virus production, the poor efficacy MM estimates an infected SIAT cell will cause the infection of only 3–5 other cells (*R*_0_).

In contrast, because the infection resistance MM relies on its resistance mechanism to arrest infection progression, an upper bound could not be determined for the duration of virus production by infected cells (*τ*_*I*_) in that MM, nor consequently for the basic reproductive number (*R*_0_), which is proportional to *τ*_*I*_. Instead of a short period of virus production, the infection resistance MM estimates cells continue to produce RSV for at least 10 days, but uninfected cells become resistant to infection ∼4.5 days post-infection (*τ*_*F*_).

The estimate for the duration of the eclipse phase in RSV-infected SIAT cells, 3 –4days, is remarkably longer than the 6–10 h eclipse phase routinely estimated for influenza A virus infections in vitro [17]. The infecting time (*t*_inf_), i.e. approximately the time it takes for a newly infected cell to cause its first infection of one other, is ∼5–10 days for RSV-infected SIAT cells, compared to 30 min–2 for influenza A virus-infected cells. Another interesting quantity is the rate at which RSV-infected SIAT cells cause secondary infections (*R*_0_/*τ*_*I*_). It is a term that takes into consideration the rate of virus production by an infectious cell, and the competing effect of the rate at which the virus progeny causes infection versus the rate at which it loses infectivity (see Methods for details). In simple terms, the infection rate corresponds to a count of individual cell infections caused per hour of virus production by a single infectious cell. Whereas a seasonal influenza A (H1N1) virus-infected cell would cause ∼20–30 new infections per hour, about 600 per day [17], we estimate a RSV-infected SIAT cell causes 1–6 infections per day.

## Discussion

Many aspects of RSV infection kinetics, including the determinant factor(s) which make RSV infectious, the manner in which this infectivity is lost or degrades over time, and the mechanism of dissemination and spread of RSV infection through a cell culture or the human respiratory tract, remain unknown or poorly resolved. Herein, using mathematical analyses and modelling techniques to dissect data from *in vitro* RSV infections in SIAT cells, we tackled some of these questions.

While a number of viruses (influenza, hepatitis C, HIV) lose infectivity following an exponential decay, it does not appear to be the case with RSV. Given our experimental data, we found that while an exponential decay correctly captures the rate at which RSV virions lose vRNA integrity, it provides the worst recapitulation of the observed decay in RSV infectivity of all MMs explored herein. In fact, all other MMs considered consistently and statistically significantly establish that the rate of loss of RSV infectivity slows down over time. This type of decay is typical of defective production or release, with defective virions failing early post-release, and with the decay rate decreasing over time as the population of defective virions thins out. In 2013, Liljeroos et al. liljeroos13 used electron cryotomography to visualize the configuration of F proteins on the surface of RSV virions during budding and release. They found that while most RSV filaments originally bud covered in F proteins in their pre-trigger (most infectious) state, imperfect budding or bud pinching led to a loss of the filament-like virion structure which triggered F proteins into their post-fusion configuration (least infectious state). This is consistent with an early rapid loss of infectivity from imperfectly budded virions, which decreases over time as these virions deform, leaving only the better formed, and therefore more infectious, filamentous virions.

Within the scope of this study, it was not possible to determine which mathematical modelling framework would be most appropriate to describe said process. This issue needs to be resolved independently before further full-scale RSV infection modelling can proceed reliably. In future work, we plan to collect more extensive mock-yield data—especially since the data herein is based on a single biological replicate—to ensure that we can: (1) identify the correct process/model for RSV infectivity loss; and (2) find an elegant and efficient way to implement it mathematically as part of a larger *in silico* RSV infection MM. Experimentally, more frequent data sampling would be required to better capture the early, more rapid decay kinetics which differed between the two virus population MM and the other MMs considered. Many more biological replicates than the single replicate considered here would also be required to ensure the decreasing infectivity decay rate is robust and reproducible, and to evaluate the extent to which the decay rate and its rate of decrease over time are consistent between experiments. Exploring how varying the initial virus concentration in the assay affects the decay rate would also serve to evaluate the dose dependence of the neutralization effect predicted by the mass-action and self-neutralization MMs considered herein. Additional work would also be needed to more definitively link F protein conformation to virion infectivity, and compare variants of the RSV F protein, e.g. from A Long, A2, and line 19 [29, 30].

The mechanisms by which RSV propagates in different cell cultures (e.g., via free virus in medium versus direct cell-to-cell infection) is an important consideration in correctly characterizing RSV properties and antiviral efficacy in such a way that *in vitro* findings will reliably translate *in vivo*. For example, it is not clear to what extent syncytia or other modes of direct cell-to-cell RSV transmission play a role in RSV infection *in vivo* [22, 27]. The need to study and characterize RSV infection kinetics in a number of different cell cultures is crucial to map the range of behaviours (mode of infection spread, intracellular immunity) and determine how these differences modulate the experimentally estimated antiviral activity. An understanding of the role of these differences will enable the identification of a cell culture in which infection kinetics is more akin to that observed *in vivo*.

**Fig 7:**
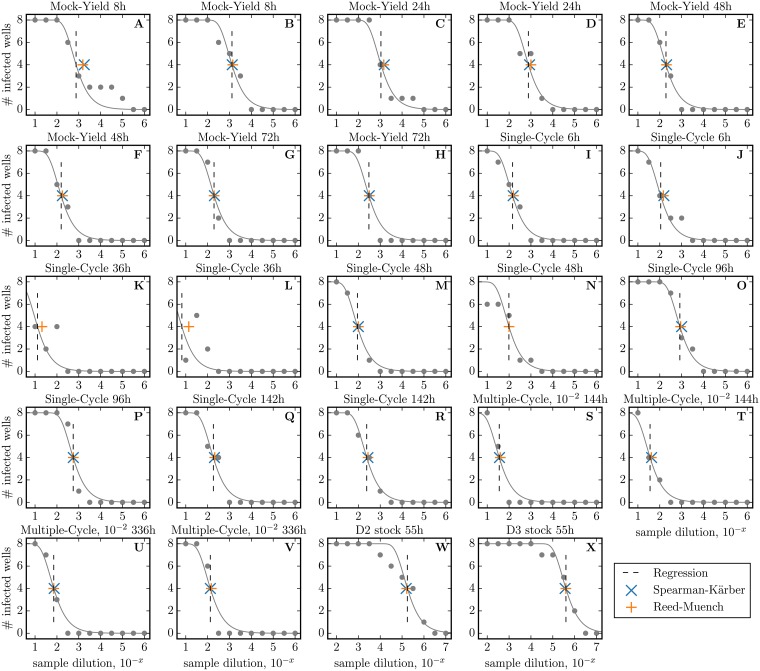
Calculation of the TCID_69_ and TCID_50_. The vertical dashed line indicates the sample dilution corresponding to 1 TCID_50_, i.e. an estimate of the dilution at which 4/8 wells are infected. This dilution is determined using a least-square regression of Eq (1) to the # of infected wells (grey line). From this TCID_50_, we compute TCID_69_ = TCID_50_ × ln(2). The TCID_50_ obtained using the Spearman-Kärber (cross) and Reed-Muench (plus) methods are also shown for comparative purposes.

The experimental data collected as part of the work conducted herein suggest that HEp-2 and SIAT cells are susceptible to the same aspect of what makes RSV infectious, i.e. RSV lose infectivity in SIAT and HEp-2 cells at comparable rates, and virions that have high (or low) infectivity in HEp-2 also do in SIAT cells. However, SIAT are far less susceptible to infection by RSV than HEp-2 cells: we found that a given RSV sample will typically be ∼ 100× more infectious in HEp-2 than in SIAT cells. Furthermore, infectious RSV titers below ∼10^4^ PFU/mL in HEp-2 cells were not quantifiable by tissue culture infectious dose (TCID_69_, Fig 7) assay in SIAT cells. The lesser susceptibility of SIAT to RSV infection means that many of the infections progressed extremely slowly, and in some cases virus production by infected SIAT cells was possibly insufficient to propagate the infection beyond the cells infected by the initial inoculum.

The RSV infection data allowed us to further test hypotheses regarding RSV infection kinetics *in vitro* and the underlying MM parameters controlling its replication and spread in SIAT cells. Our analysis shows conclusively that RSV-infected SIAT do indeed produce infectious virus, but that infection is largely limited to the cells infected by the initial inoculum, with infection proceeding essentially as a single-cycle (SC) infection. The hypothesis that RSV infection is progressing slowly due to poor infection efficacy was rejected in favour of the hypothesis that a potent resistance to infection, arising around 4.5 days post infection, could be acting to suppress cell infection by RSV.

**Fig 8:**
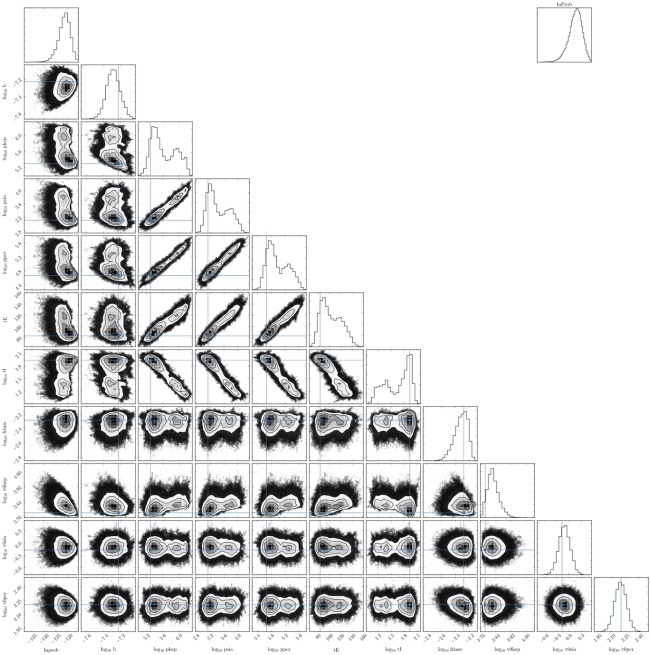
Pairwise PostPLDs for the poor infection efficacy MM.

**Fig 9:**
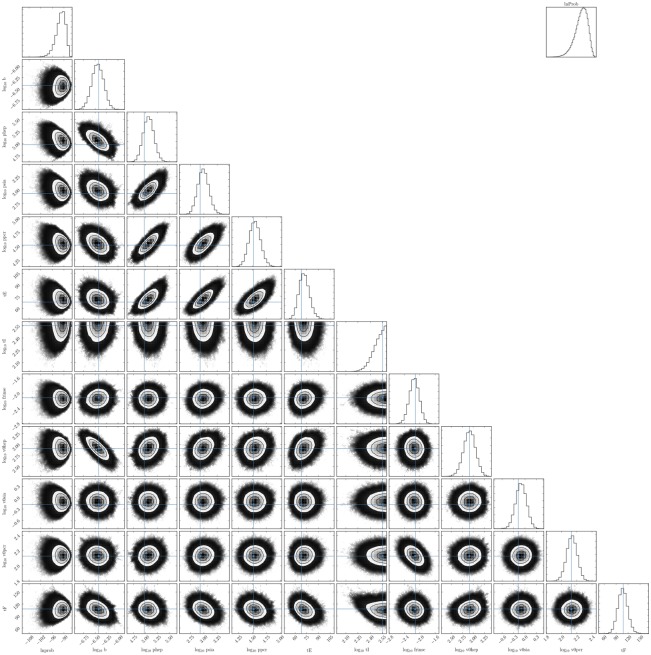
Pairwise PostPLDs for the infection resistance MM.

The estimate obtained here for the timing of the onset of infection resistance can be tested in future work against the kinetics of defective interfering RSV concentration or possible antiviral candidates (e.g., IFN in SIAT infected with RSV). We found that measurements of RSV concentration in the supernatant over more than 2 weeks of infection, but also more frequently, would be needed to observe the timing of RSV concentration decay in the assay. This would help identify the duration of virus production by RSV-infected cells (*τ*_*I*_), would improve the accuracy of several other parameters correlated to this parameter (see Figs 8 and 9), and could permit a greater discrimination between (or the elimination of) the poor efficacy or infection resistance MM hypotheses.

Despite their poor RSV yields, we believe SIAT cells are better suited than HEp-2 to study RSV infection kinetics *in vitro*. While RSV infection proceeds with great difficulty in SIAT cells, this is likely more in line with the very slow progression of RSV infection *in vivo*. Bagga et al. [31] have shown that adults experimentally infected will see their viral titer peak 5–6 days post inoculation with RSV compared to 2–3 dpi for those infected with the influenza A virus. This slower infection kinetics for RSV compared to the influenza virus is well recapitulated in the SIAT cell culture infection experiments (see Fig 6 herein), whereas infection proceeds faster in HEp-2 cells. For example, see Fig 1 in [21] which compares RSV infection kinetics in HEp-2 and MDCK cells, wherein infectious RSV yield in HEp-2 cells peaks 1–2 day earlier despite the 100-fold smaller RSV inoculum used by the authors to infect the HEp-2 cells. The HEp-2 infection kinetics would likely have been even more rapid if an equivalent RSV inoculum had been used to infect both cell cultures [32]. The slow infection progression in SIAT could be due to the different manner in which RSV infection spreads, e.g. more via cell walls and syncytia in the non-polarized HEp-2 for example, versus by free virus at the apical surface in the polarized MDCK-SIAT cells [21], and/or perhaps due to the absence of biologically relevant factors mediating infection resistance in HEp-2 cells infected with RSV.

## Conclusion

The careful, quantitative, mathematical analysis of *in vitro* RSV infection kinetics performed here has allowed us to test hypotheses regarding the mechanisms driving (1) RSV loss of infectivity; and (2) the slow or arrested RSV infection kinetics. It has enabled us to identify key properties of RSV infections which are poorly understood yet critical to correctly interpret RSV infection kinetics to design appropriate vaccines and antivirals. Based on these findings, we conclude that *there is a need to resolve mechanism(s) behind RSV loss of infectivity*. These mechanisms are still largely unresolved and yet are a critical consideration in correctly interpreting any RSV infection kinetics. In particular, there is mounting evidence that infectivity of RSV could relate to RSV morphology and that this same morphology (in particular, the state of the pre- vs post-trigger fusion protein) has important implications for adaptive immunity (notably humoral immunity) to RSV and rational design of vaccines to target the correct subpopulation of RSV virions [13, 33]. There is, therefore, an urgency, in our opinion, to resolve the question of RSV infectivity, and its correspondence to RSV morphology and antigenicity, to ensure safe and effective vaccine and antiviral design.

## Methods

### Virus and cells

The RSV A Long (ATCC VR-26) strain was used in all experiments. For all RSV infection experiments, RSV was used to infect ST6GalI-transfected Madin-Darby canine kidney cells (MDCK*α*2, 6, hereafter SIAT) which are transfected to express *α*-2, 6 sialic acid receptors on their surface at levels consistent with human respiratory tract epithelium [34]. These cells were kindly provided by Prof. Yoshihiro Kawaoka, University of Wisconsin, USA. Infectious virus sampled from the infected SIAT supernatant was quantified both via 69% tissue culture infectious dose (TCID_69_) on SIAT, and via plaque forming units (PFU) on human HeLa contaminant carcinoma epithelial cells (ATCC CCL-23), hereafter HEp-2.

### Mock-yield assay

A 5 mL solution containing 10^4.5^ TCID_69_/mL of RSV A Long viruses in 5% FBS MEM was placed in a T-25 flask without cells at 37°C in a 5% CO_2_ incubator. After 8 h, 24 h, 48 h and 72 h of incubation, 1 mL of the virus-containing media was collected from the flask. Some of the fresh media was deposited onto SIAT and HEp-2 cell cultures to quantify infectious virus concentration immediately upon collection in duplicate. The remainder was quickly frozen in liquid nitrogen and stored at −80°C to be later quantified in duplicate for total virus concentration, as described below. As such, this experiment was conducted as a single biological sample, quantified in duplicate in three different ways. These data are provided in S1 File.

### Infection assays

Five (5) days prior to infection, SIAT cells were seeded in 12-well plates, giving them sufficient time to reach confluence. No infection was carried out in HEp-2 cells, although both HEp-2 and SIAT cells were used to quantified the virus titer sampled from the supernatant of the infected SIAT wells, as described below. All infection data are provided in S1 File.

For the RSV infection experiment at the highest inoculum dose, the confluent SIAT cells were infected in duplicate with 1 mL of an inoculum containing 10^4.6^ TCID_69_/well of RSV A Long, representing a multiplicity of infection (MOI) of ∼0.05 TCID_69_/cell. After 6 h in an incubator at 5% CO_2_, the infecting medium was removed, the cells were rinsed with 1× HBSS twice, and 2 mL of 5% FBS MEM virus-free medium was added to the cultures which were placed back in the 5% CO_2_ incubator. At 8, 12, 16, 24, 36, 48, 72, 96, 120, 142, and 168 h post-infection, the supernatants of 2 duplicate wells were harvested. For the two RSV infections at the lower inoculum dose, the confluent SIAT cells were infected in duplicate with 2 mL of an inoculum containing either 10^0.74^ or 10^1.74^ TCID_69_/well of RSV A Long, representing a MOI of ∼ 10^−6^ or 10^-5^ TCID_69_/cell, respectively, and placed in an incubator at 5% CO_2_. The infecting medium was never rinsed, and the medium was not replaced over the course of these infections. At 12, 16, 24, 36, 48, 72, 96, 120, 144, 168, and 336 h post-infection, the supernatants of 2 duplicate wells were harvested.

Supernatants harvested over the course of the three separate infection experiments were, immediately upon harvesting, placed fresh on SIAT and HEp-2 cells for infectious virus quantitation, and the rest was frozen at −80°C to be later quantified in duplicate for total virus concentration, as described below.

### Quantification of total virion concentration

Quantitative real-time, reverse transcription polymerase chain reaction (qRT-PCR) was performed after one freeze-thaw cycle as previously described [35]. The measurements obtained via qRT-PCR herein are reported in relative units of viral RNA equivalents (vRNAe) which have been rescaled using a standard curve based on the PFU concentration of the standard, rather than on the number of RSV RNA segments it contains.

### Quantification of infectious virus in HEp-2 cells

Plaque assays were performed as previously described [36, 37]. Briefly, four 10-fold dilutions (ranging from undiluted to 10^−3^) of fresh viral infected supernatants were prepared and placed in triplicate in 12-well culture plates containing 80% confluent HEp-2 cells. Supernatants were incubated for 1 h and then overlaid with 1 mL of pre-warmed 0.75% methylcellulose-containing growth media, without rinsing the initial inoculum. Plates were incubated for five days at 37°C (5% CO_2_), fixed with formalin, and stained with hematoxylin and eosin for plaque counting and morphology observation. Infectious RSV concentration in the range 10^0.48^–10^8.67^ PFU/mL were theoretically quantifiable in this assay.

### Quantification of infectious virus in SIAT cells

Confluent cells in 96-well plates were infected with eleven 10^0.5^-fold dilutions of fresh viral supernatant ranging from 1:10^1^ to 1:10^6^. After 6 h in an incubator at 5% CO_2_, the supernatants were discarded, the cell monolayers were washed with 1× HBSS twice, and 200 μL of 5% FBS MEM virus-free medium was added. Plates were incubated for five days at 37°C (5% CO_2_), fixed with formalin, tagged with anti-RSV F and anti-RSV NP antibodies (Millipore, MAB8262, MAB8598, MAB8599, MAB858-3B) conjugated with alkaline phosphatase anti-mouse IgG (Sigma-Aldrich, A3562), and stained with vector blue substrate kit (VECTOR, SK-5300) for visualization. The infectious virus concentration was determined using a least-square regression to the following expression

$$N_{\text{infected}}=N_{\text{wells/dilution}}[1-\text{exp}(-\frac{K_{0}}{D})]$$ (1)

where *N*_infected_ is the number of positive (infected) wells out of the *N*_wells/dilution_ (= 8 wells) inoculated at each dilution *D* ∈ [10^1^, 10^6^], and *K*_0_ is the infectious virus concentration in the undiluted sample [38]. Quantity *K*_0_ is equivalent to PFU as it corresponds to the dilution at which 69.3% of the wells are infected (1 − e^−1^ = 0.693 or ∼5/8 wells, when *D* = *K*_0_), or 1 TCID_69_ ≈ 1*PFU*. The more common TCID_50_, the dilution at which 50% of wells are infected, is such that ${\text{TCID}}_{69}={\text{TCID}}_{50}\times \text{ln}(2)(1-{\text{e}}^{-K_{0}/D}=0.5\;\text{or}\;K_{0}=-\text{ln}(2)D)$. In Fig 7, we compare our TCID_69_/ln(2) extracted via least-square regression against the TCID_50_ obtained using the Reed-Muench and Spearman-Kärber methods and find consistent values. The limits of detection of the assay was approximately 10^2^−10^7^ TCID_69_/mL.

### Mathematical models for RSV loss of infectivity

The exponential MM is

$$\frac{\text{d}V}{\text{d}t}=-cV\;V\left(t\right)=V_{0}\text{exp}\left[-ct\right]\;.$$ (2)

The Weibull MM, which reduces to the exponential MM when *k* = 1, is

$$\frac{\text{d}V}{\text{d}t}=-\left[k{\left(ct\right)}^{k-1}\right]cV\;V\left(t\right)=V_{0}\text{exp}\left[-{\left(ct\right)}^{k}\right]\;.$$ (3)

The mass-action MM represents the neutralization of virus *V* by an unknown compound *N* which is initially present in proportion *f* = *N*_0_/*V*_0_, but is consumed as *V* gets neutralized, such that

$$\begin{array}{l} \frac{\text{d}V}{\text{d}t}=-kV\left[\frac{N_{0}-\left(V_{0}-V\right)}{V_{0}}\right]\;V\left(t\right)=\frac{\left(1-f\right)V_{0}}{1-f\text{exp}\left[-\left(1-f\right)kt\right]}\;.\\ \frac{\text{d}V}{\text{d}t}=-kV\left[f-1+\frac{V}{V_{0}}\right] \end{array}$$ (4)

The self-neutralizing MM, which corresponds to the mass-action MM with *f* = 1, is

$$\frac{\text{d}V}{\text{d}t}=-cV\frac{V}{V_{0}}\;V\left(t\right)=\frac{V_{0}}{1+ct}\;.$$ (5)

The two population MM represents two distinct populations of virions of which initially a fraction *f*_low_ is of type *V*_low_ which degrades at slower rate *c*_low_, and the remainder, (1 − *f*_low_), are of type *V*_high_ which degrade more rapidly at rate *c*_high_ > *c*_low_, such that

$$\begin{array}{llll} \frac{\text{d}V_{\text{high}}}{\text{d}t}=-c_{\text{high}}V_{\text{high}} & & & V\left(t\right)=V_{\text{high}}\left(t\right)+V_{\text{low}}\left(t\right)\\ \frac{\text{d}V_{\text{low}}}{\text{d}t}=-c_{\text{low}}V_{\text{low}} & & & V\left(t\right)=V_{0}\left[f_{\text{low}}{\text{e}}^{-c_{\text{low}}t}+\left(1-f_{\text{low}}\right){\text{e}}^{-c_{\text{high}}t}\right]\;. \end{array}$$ (6)

The two age class MM represents two states of infectivity in which virions can find themselves, with virions initially being all found in the highly infectious *V*_high_ state, from which they exponentially decay at rate *r*_pre_ into the *V*_low_ state which is $\frac{r_{\text{pre}}}{r_{\text{post}}}\times$ less infectious (*r*_pre_ > *r*_post_), until ultimately they lose all infectivity at exponential rate *c*_low_.

$$\begin{array}{llll} \frac{\text{d}V_{\text{high}}}{\text{d}t}=-r_{\text{pre}}V_{\text{high}} & & & V\left(t\right)=V_{\text{high}}\left(t\right)+V_{\text{low}}\left(t\right)\\ \frac{\text{d}V_{\text{low}}}{\text{d}t}=r_{\text{post}}V_{\text{high}}-c_{\text{low}}V_{\text{low}} & & & V\left(t\right)=V_{0}{\text{e}}^{-r_{\text{pre}}t}\left[1-\frac{r_{\text{post}}}{r_{\text{pre}}-c_{\text{low}}}\left(1-{\text{e}}^{\left(r_{\text{pre}}-c_{\text{low}}\right)t}\right)\right]\;. \end{array}$$ (7)

It is easy to show that the two population and two age class MMs are mathematically equivalent,

$$\begin{array}{cc} \frac{V(t)}{V_{0}} & =[e^{-r_{\text{pre}}t}-\frac{r_{\text{post}}}{r_{\text{pre}}-c_{\text{low}}}e^{-r_{\text{pre}}t}+\frac{r_{\text{post}}}{r_{\text{pre}}-c_{\text{low}}}e^{-r_{\text{pre}}t}e^{(r_{\text{pre}}-c_{\text{low}})t}]\\ & =[f_{\text{low}}e^{-c_{\text{low}}t}+e^{-c_{\text{high}}t}-f_{\text{low}}e^{-c_{\text{high}}t}]\\ \frac{V(t)}{V_{0}} & =[e^{-r_{\text{pre}}t}-\frac{r_{\text{post}}}{r_{\text{pre}}-c_{\text{low}}}e^{-r_{\text{pre}}t}+\frac{r_{\text{post}}}{r_{\text{pre}}-c_{\text{low}}}e^{-c_{\text{low}}t}]\\ & =[e^{-c_{\text{high}}t}-f_{\text{low}}e^{-c_{\text{high}}t}+f_{\text{low}}e^{-c_{\text{low}}t}]\;, \end{array}$$ (8)

when *c*_high_ = *r*_pre_, *f*_low_ = *r*_post_/(*r*_pre_ − *c*_low_), and *c*_low_ is the same in both MMs.

### Mathematical model for RSV infection kinetics

The *in vitro* RSV infection experiments were simulated numerically using an age-structured, ordinary differential equation (ODE) model, introduced and described previously [18, 39]. The ODE MM, described the time course of *in vitro* infection of SIAT cells as

$$\begin{array}{cc} \frac{dT}{dt} & =-\beta TV_{\text{HEp}-2}\\ \frac{dE_{1}}{dt} & =\beta TV_{\text{HEp}-2}-\frac{n_{E}}{\tau_{E}}E_{1}\\ \frac{dE_{i}}{dt} & =\frac{n_{E}}{\tau_{E}}E_{i-1}-\frac{n_{E}}{\tau_{E}}E_{i}\;\;\;\text{for}\;i=\left(2,\cdots ,n_{E}\right)\\ \frac{dI_{1}}{dt} & =\frac{n_{E}}{\tau_{E}}E_{n_{E}}-\frac{n_{I}}{\tau_{I}}I_{1}\\ \frac{dI_{j}}{dt} & =\frac{n_{I}}{\tau_{I}}I_{j-1}-\frac{n_{I}}{\tau_{I}}I_{j}\;\;\;\text{for}\;j=\left(2,\cdots ,n_{I}\right) \end{array}$$ (9)

wherein a population of uninfected target cells (*T*) become infected by infectious RSV (*V*_HEp-2_) at rate *β*. The newly infected cells first enter the eclipse phase ($E_{i=1,...,n_{E}}$) wherein they initiate intracellular virus replication. An average time *τ*_*E*_ later, intracellular virus replication has progressed to the point that cells leave the eclipse phase to enter the infectious phase ($I_{j=1,...,n_{I}}$) and begin releasing virions. An average time *τ*_*I*_ after they begun releasing virus, the infectious cells will cease production and undergo apoptosis. The MM considers both infectious RSV, experimentally quantified either on SIAT (*V*_SIAT_, measured in TCID_69_/mL) or HEp-2 cells (*V*_HEp-2_, measured in PFU/mL), and total virion concentration quantified by qRT-PCR (*V*_PCR_, measured in units proportional to vRNA count, i.e. vRNA equivalents/mL). These 3 different ways to quantify RSV track 3 different RSV particle subspecies released by infectious cells into the supernatant over the course of the *in vitro* infection of SIAT cells. They are represented mathematically as

$$\begin{array}{cc} \frac{dV_{\text{PCR}}}{dt} & =p_{\text{PCR}}\sum_{j=1}^{n_{I}}I_{j}-c_{\text{PCR}}V_{\text{PCR}}\\ \frac{dV_{\text{SIAT}}^{\text{high}}}{dt} & =p_{\text{SIAT}}\sum_{j=1}^{n_{I}}I_{j}-r_{\text{pre}}V_{\text{SIAT}}^{\text{high}}\\ \frac{dV_{\text{SIAT}}^{\text{low}}}{dt} & =r_{\text{post}}V_{\text{SIAT}}^{\text{high}}-c_{\text{inf}}V_{\text{SIAT}}^{\text{low}}\\ \frac{dV_{\text{HEp}-2}^{\text{high}}}{dt} & =p_{\text{HEp}-2}\sum_{j=1}^{n_{I}}I_{j}-r_{\text{pre}}V_{\text{HEp}-2}^{\text{high}}\\ \frac{dV_{\text{HEp}-2}^{\text{low}}}{dt} & =r_{\text{post}}V_{\text{HEp}-2}^{\text{high}}-c_{\text{inf}}V_{\text{HEp}-2}^{\text{low}} \end{array}$$ (10)

wherein total (*V*_PCR_) and infectious (*V*_SIAT_ and *V*_HEp-2_) RSV are released at constant rates *p*_PCR_, *p*_SIAT_, and *p*_HEp-2_, respectively, into the supernatant by infectious SIAT cells. The concentration of total virus (vRNAe/mL) in the medium degrades and is lost at a rate of *c*_PCR_. Since RSV loss of infectivity does not appear to proceed at an exponential rate, a two age class MM was considered instead wherein $V_{\text{SIAT}}^{\text{high}}$ and $V_{\text{HEp}-2}^{\text{high}}$ are the highly infectious form of the virus, which rapidly decay at rate *r*_pre_ into the much less infectious form of the virus, $V_{\text{SIAT}}^{\text{low}}$ and $V_{\text{HEp}-2}^{\text{low}}$, which ultimately completely lose infectivity at rate *c*_inf_. The more infectious (high) form of the virus, is *r*_pre_/*r*_post_ times more infectious than the less infectious (low) form. In the equations for *T* and *E*_1_ in Eq (9), and in comparing the MM-predicted curve to the experimental data, $V_{\text{HEp}-2}=V_{\text{HEp}-2}^{\text{high}}+V_{\text{HEp}-2}^{\text{low}}$ and $V_{\text{SIAT}}=V_{\text{SIAT}}^{\text{high}}+V_{\text{SIAT}}^{\text{low}}$.

To account for the plateau in the total virus concentration, a crude IFN-like cellular antiviral response was also considered, referred to as the infection resistance MM. Infection resistance in this MM is achieved by simply blocking cell infection after some time, *τ*_*F*_, such that

$$\text{virus}\;\text{infectivity}=\{\begin{array}{cc} \beta & \text{,}\;t<\tau_{F}\\ 0 & \text{,}\;t\geq \tau_{F} \end{array}\;.$$ (11)

This expression is meant to capture the sudden onset of an IFN-like antiviral response granting resistance to infection to all cells remaining uninfected by time *τ*_*F*_ post-infection.

### Parameter estimation for RSV infection MMs

The RSV infection kinetics MM, Eqs (9) and (10), and its variant with an IFN-like antiviral response, Eqs (9)–(11), simultaneously produces the 9 different kinetic curves: one for each of the 3 different measures of RSV subspecies in the sampled supernatant (vRNAe, PFU and TCID_69_) for each of the 3 different MOIs used to experimentally infect SIAT cells, using a single set of parameters. RSV decay parameters (*c*_PCR_, *c*_inf_, *r*_pre_, *r*_post_) were fixed to the values reported in Table 1. The number of eclipse and infectious phases traversed by SIAT cells upon RSV infection were fixed to *n*_*E*_ = 6 and *n*_*I*_ = 60, respectively. The value *n*_*I*_ = 60 was chosen because the sustained viral production in the infection assay (Fig 4) is consistent with a normally-distributed infectious (virus-producing) duration and for *n*_*I*_ > 30 or so, the infectious phase is normal-like but not sensitive to the precise value of *n*_*I*_ [40, 41]. The value *n*_*E*_ = 6 was chosen because in preliminary parameter fitting, values of *n*_*E*_ ∈ [3, 7] were favoured over several infection MMs variants considered. This leaves a total of 4 initial conditions (*V*_PCR_ (0), *V*_HEp-2_ (0), *V*_SIAT_ (0), *V*_post-rinse_/*V*_pre-rinse_) and 6–7 parameters (*p*_PCR_, *p*_HEp-2_, *p*_SIAT_, *β*, *τ*_*E*_, *τ*_*I*_, *τ*_*F*_) to be estimated from the experimental RSV infection data.

The 10 or 11 parameters of the infection MMs were estimated through a MCMC process with the emcee python library [42]. The PostPLDs correspond to 300 chains of 6000 steps each (1,800,000 parameter sets), after a burn-in of 1,800 steps (poor efficacy MM) or 6,000 steps (infection resistance MM). The likelihood of accepting a particular parameter set proposition ($\vec{p}$) was computed as $L\left(\vec{p}\right)={\text{exp}}^{-\text{SSR}(\vec{p})}$, where $\text{SSR}(\vec{p})$ is the overall sum of squared residuals between the MM-generated curves and the 9 data sets shown in Fig 4. The extent to which any one individual MM parameter is identifiable is indicated by its 95% CR provided in Table 2. Figs 8 and 9, produced using the python corner library [43], provide pairwise parameter PostPLDs showing correlations between pairs of parameters in each MM considered. The more significant parameter correlations were between the rates of RSV production (*p*_PCR_, *p*_HEp-2_, *p*_SIAT_), and the durations of the eclipse (*τ*_*E*_) and virus production (*τ*_*I*_) phases. This is in part because the experimental data is not always sufficient to distinguish between having 3× more virus produced over 1/3 the time versus having 1/3× as many virus produced over a 3× longer period.

Virus infectivity (*β*) showed little to no correlation to virus production rates (*p*_PCR_, *p*_HEp-2_, *p*_SIAT_) in the present work. However, it is important to remember that even when uncorrelated, these parameters which carry units of virus (TCID_69_, PFU, vRNAe), are of limited meaning or value individually because they are relative rather than absolute quantities [17]. For an identical virus sample, measures of infectious virus are expected to vary as a function of the cell culture in which infectious virions are quantified, and several other experimental procedural details (e.g., whether samples are frozen prior to the measurement [44]). By taking their product, *β* ⋅ *p*_HEp-2_, it is possible to eliminate their relative units of virus measurement. The infecting time, computed as $t_{\text{inf}}=\sqrt{2/(\beta \cdot p_{\text{HEp}-2})}$, is a useful way to express this product, and it corresponds approximately to time between the start of virus production by a newly infected cell and the first infection it causes [45].

Infection kinetics depends not only on virus produced and their infectivity, but also on the competing effect of the rate at which virions cause infection versus the rate at which they lose infectivity. For this reason, we rely on a measure we call the infection rate, a parameter which accounts for all these different competing kinetics to yield a single number, which is unaffected by the relative virus units as these cancel themselves out as part of the calculation of this quantity. The infection rate is related to the basic reproductive number, i.e., the number of secondary cells infected by a single RSV-infected cell over its infectious lifespan. Specifically, the infection rate corresponds to the basic reproductive number divided by the infectious cell lifespan (*R*_0_/*τ*_*I*_). For our MM above, the infection rate is given by

$$\frac{R_{0}}{\tau_{I}}=\underset{\text{Two}\;\text{age}\;\text{classes}}{\underset{︸}{\overset{\text{Exp}}{\overset{︷}{\frac{p\beta T_{0}}{c_{\text{inf}}}}}\cdot \frac{r_{\text{post}}+c_{\text{inf}}}{r_{\text{pre}}}}}$$ (12)

when using the exponential (Exp) or the two age class MM for RSV loss of infectivity.

### Calculation of *p*-values

Throughout this work, *p*-values are provided to quantify the statistical significance of various statements, e.g. model A performed better than B in terms of AIC_C_; or model A estimates a higher value than model B for some parameter. In all these statements, the quantities being compared correspond to two different MCMC-estimated, PostPLDs. We compute the *p*-value as the fraction of time the statistical statement, e.g. “A is greater than B”, is true out of 6,000,000 pairwise comparisons, wherein each pair is formed by drawing one value from each the model A and B PostPLDs for the quantity being compared, sampled at random with replacement. This is similar to the Mann-Whitney U test but yields more conservative, i.e. less significant (larger) *p*-values, because our method assigns a score of zero, rather than 0.5, for ties (i.e., when A = B).

## Supporting information

S1 File Experimental data. All data analyzed within this manuscript are provided as a spreadsheet.
(XLS)
